# Opening of Large Institutions of Higher Education and County-Level COVID-19 Incidence — United States, July 6–September 17, 2020

**DOI:** 10.15585/mmwr.mm7001a4

**Published:** 2021-01-08

**Authors:** Andrew J. Leidner, Vaughn Barry, Virginia B. Bowen, Rachel Silver, Trieste Musial, Gloria J. Kang, Matthew D. Ritchey, Kelly Fletcher, Lisa Barrios, Eric Pevzner

**Affiliations:** ^1^CDC COVID-19 Response Team; ^2^Geospatial Research, Analysis, and Services Program, CDC/ATSDR, Atlanta, Georgia; ^3^HHS COVID-19 Joint Coordination Cell.

During early August 2020, county-level incidence of coronavirus disease 2019 (COVID-19) generally decreased across the United States, compared with incidence earlier in the summer (*1*); however, among young adults aged 18–22 years, incidence increased (*2*). Increases in incidence among adults aged ≥60 years, who might be more susceptible to severe COVID-19–related illness, have followed increases in younger adults (aged 20–39 years) by an average of 8.7 days (*3*). Institutions of higher education (colleges and universities) have been identified as settings where incidence among young adults increased during August (*4*,*5*). Understanding the extent to which these settings have affected county-level COVID-19 incidence can inform ongoing college and university operations and future planning. To evaluate the effect of large colleges or universities and school instructional format* (remote or in-person) on COVID-19 incidence, start dates and instructional formats for the fall 2020 semester were identified for all not-for-profit large U.S. colleges and universities (≥20,000 total enrolled students). Among counties with large colleges and universities (university counties) included in the analysis, remote-instruction university counties (22) experienced a 17.9% decline in mean COVID-19 incidence during the 21 days before through 21 days after the start of classes (from 17.9 to 14.7 cases per 100,000), and in-person instruction university counties (79) experienced a 56.2% increase in COVID-19 incidence, from 15.3 to 23.9 cases per 100,000. Counties without large colleges and universities (nonuniversity counties) (3,009) experienced a 5.9% decline in COVID-19 incidence, from 15.3 to 14.4 cases per 100,000. Similar findings were observed for percentage of positive test results and hotspot status (i.e., increasing among in-person–instruction university counties). In-person instruction at colleges and universities was associated with increased county-level COVID-19 incidence and percentage test positivity. Implementation of increased mitigation efforts at colleges and universities could minimize on-campus COVID-19 transmission.

The National Center for Educational Statistics’ Integrated Postsecondary Education Data System (*6*) was used to identify not-for-profit baccalaureate degree–granting colleges and universities enrolling ≥20,000 full-time and part-time students. Colleges and universities that enrolled <20,000 students or were considered for-profit were excluded. Fall class start dates and instructional formats on the first day of scheduled classes were abstracted from college and university websites during early September 2020. Counties with large colleges and universities were assigned the start date and instructional format of the school. If a county contained multiple large colleges or universities with different start dates, the earliest start date and corresponding instructional format was assigned. If a county contained multiple large schools with the same start date but different instructional formats, then in-person instruction was assigned. Among 133 counties with large colleges and universities (university counties),^†^ the 101 (76%) in which classes started from July 27 to August 28 were included in the analysis (i.e., 32 were excluded because they included institutions that started on or after August 29 and had insufficient data for the 21 days after the start of classes at the time of analysis). County-level mean estimates of COVID-19 incidence,^§^ testing rates, percentage test positivity,^¶^ and hotspot status** were compared for university counties with remote-instruction, in-person–instruction, and nonuniversity counties during the 21 days before and after the start of classes.

For all analyses, mean county population size, full-time student enrollment size, urban-rural classifications (large central metro, large fringe metro, medium metro, small metro, micropolitan, and noncore), and COVID-19 outcomes are reported and stratified by county university status and instructional format. The COVID-19 outcomes included incidence and testing rates per 100,000 population, test positivity by SARS-CoV-2 reverse transcription–polymerase chain reaction (RT-PCR) testing, and the percentage of counties identified as hotspots for ≥1 day during the observation periods. COVID-19 outcomes were reported as means for the 21 days before and after the class start date. Absolute differences (i.e., percentage point differences) are described for percentage-based measures (test positivity and hotspot detection) and relative changes described for rate-based measures (testing rate and incidence). Seven-day moving averages for testing rates, percentage test positivity, and incidence are presented as trends over the observation period (day –21 to day +21). In an unmatched analysis, remote-instruction and in-person instruction university counties were compared with nonuniversity counties. Nonuniversity counties were assigned the median start date of university counties. In the matched analysis, in-person–instruction university counties were matched with nonuniversity counties based on geographic proximity and population size. This analysis of 68 matched pairs was conducted to account for differences in population size, urbanicity, and geographic location between university and nonuniversity counties.^††^ Nonuniversity counties in the matched sample were assigned the start date of their matched university-county counterpart. In the matched analysis, a regression-based difference-in-difference approach^§§^ was used to quantify the impact of in-person instruction on COVID-19 incidence, with and without adjustment for transient student populations,^¶¶^ and percentage test positivity. A sensitivity analysis was conducted to explore whether students’ early return to campus might affect observed changes using day –7 as the demarcation between before and after periods. Statistical significance was set at α = 0.05. Analyses were conducted using R statistical software (version 4.0.2; The R Foundation).

Among 101 university counties (3.2% of all U.S. counties, accounting for 29.4% of the U.S. population), instructional format was remote for 22 (22%) and in-person for 79 (78%). University counties had higher mean population size and were more urban than were nonuniversity counties (Table). Before the start of school, COVID-19 testing rates at the county-level were already higher in university counties than in nonuniversity counties (Figure). Comparing the time from the start of classes through day 21 with the 21 days before classes began, mean daily testing increased 4.2% and 14.1% among remote instruction and in-person instruction university counties, respectively, and decreased 1.0% among nonuniversity counties. Mean test positivity decreased among remote-instruction university counties (absolute change = –1.8%) and nonuniversity counties (–0.6%) but increased among in-person instruction university counties (1.1%). Incidence decreased in nonuniversity counties (–5.9%) and remote-instruction counties (–17.9%), whereas, incidence increased in in-person (56.2%) university counties. The percentage of counties identified at least once as a hotspot increased for all three groups, with the highest percentage observed in in-person instruction university counties (30.4% absolute increase), followed by remote-instruction university counties (9.1%) and nonuniversity counties (1.5%).

**TABLE:** COVID-19 testing, percentage positivity, incidence, and county hotspot status among counties with and without colleges and universities,* by instructional format on the first day of the fall 2020 semester — United States, 2020

Characteristic	Unmatched analysis			Matched analysis^†^	
	University counties^§^		Nonuniversity counties	University counties	Nonuniversity counties
	Remote instruction	In-person instruction		In-person instruction	
**Total no. of counties**	**22**	**79**	**3,009**	**68**	**68**
Mean county population	1,694,739	748,544	69,574	467,187	413,460
Total no. of large colleges/universities	31	84	—	71	—
Mean college/university full-time enrollment in county**^§^**	37,769	27,451	—	27,084	—
Mean percentage full-time college/university enrollment of total county population	7.7	11.7	—	13.3	—
**Percentage of counties in each urban-rural category^¶^**					
Large central metro	59	27	1	16	9
Large fringe metro	9	13	12	13	32
Medium metro	18	28	11	32	28
Small metro	5	25	11	29	18
Micropolitan	9	8	21	9	9
Noncore	0	0	44	0	4
County COVID-19 testing rate per 100,000 population**					
Mean daily rate from day –21 to day –1^††^	308	255.0	209	256.0	216
Mean daily rate from day 0 to day 21	321	291.0	207	304.0	204
Relative change, %^§§^	4.2	14.1	−1.0	18.8	–5.6
County COVID-19 RT-PCR test percentage positivity**					
Mean from day –21 to day –1	8.1	7.8	8.7	7.5	8.6
Mean from day 0 to day 21	6.4	8.9	8.0	9.1	7.9
Absolute change, %^§§^	−1.8	1.1	–0.6	1.6	–0.8
**County COVID-19 incidence^¶¶^**					
Mean incidence from day –21 to day –1	17.9	15.3	15.3	14.3	16.9
Mean incidence from day 0 to day 21	14.7	23.9	14.4	25.5	13.6
Relative change, %^§§^	–17.9	56.2	–5.9	78.3	–19.5
County COVID-19 hotspot activity ***					
Percentage detected as a hotspot from day –21 to day –1	9.1	8.9	4.4	8.8	13.2
Percentage detected as a hotspot from day 0 to day 21	18.2	39.2	5.9	42.6	14.7
Absolute change, %^§§^	9.1	30.4	1.5	33.8	1.5

**Abbreviations:** COVID-19 = coronavirus disease 2019; RT-PCR = reverse transcription–polymerase chain reaction.

* 133 counties had institutions of higher education (large colleges or universities). Some counties (n = 32; 24%) opened on or after August 29 and were excluded from analysis. University counties are defined as counties with a large college or university. Nonuniversity counties are defined as counties without a large college or university.

^†^ University counties matched to geographically proximate comparison counties with similar population size. Matches for each university county were identified by first listing all candidate (county) matches without large colleges and universities (nonuniversity counties) that had a similar population size (± 30%) and that were located within 500 miles (805 km) of each university county. From these candidate matches the final match was selected based on closest proximity. After matching, the average distance between counties in matched university county and nonuniversity county pairs was 114 miles (183 km) with a maximum distance of 471 miles (758 km).

^§^ Colleges and universities were included in the analysis if they had ≥20,000 total enrolled students, which included full-time and part-time students. The full-time student enrollments from these included institutions were combined across each university county. The number of full-time student enrollments in the university counties ranged from 11,774 to 192,173.

^¶^ Urban-rural classifications are from the National Center for Health Statistics’ six-level urban-rural classification scheme for U.S. counties (https://www.cdc.gov/nchs/data_access/urban_rural.htm).

** Testing rates and percentage positivity for reverse transcription–polymerase chain reaction tests were obtained from COVID-19 electronic laboratory reporting data submitted by state health departments and from data submitted directly by public health, commercial, and reference laboratories.

^††^ Day –21, –1, and 21 are relative to day 0, which indicates the start date of instruction at colleges and universities for the fall 2020 semester. The nonuniversity counties were assigned the median start date in the unmatched analysis and were assigned the start date of their matched university county counterpart in the matched analysis.

^§§^ Absolute differences are described for percentage-based measures (i.e., test positivity and hotspot detection) and relative changes described for rate-based measures (i.e., testing rate and incidence).

^¶¶^ Incidence (cases per 100,000) was calculated using daily reported COVID-19 case-counts from state and county health department websites compiled by USAFacts (https://usafacts.org/).

*** Hotspot, or rapid riser, counties met all four of the following criteria, relative to the date assessed: 1) >100 new COVID-19 cases in the most recent 7 days; 2) an increase in the most recent 7-day COVID-19 incidence over the preceding 7-day incidence; 3) a decrease of no more than 60% or an increase in the most recent 3-day COVID-19 incidence over the preceding 3-day incidence; and 4) a ratio of 7-day incidence to 30-day incidence exceeding 0.31. In addition to those four criteria, hotspots met at least one of the following criteria: 1) >60% change in the most recent 3-day COVID-19 incidence or 2) >60% change in the most recent 7-day incidence.

**FIGURE:**
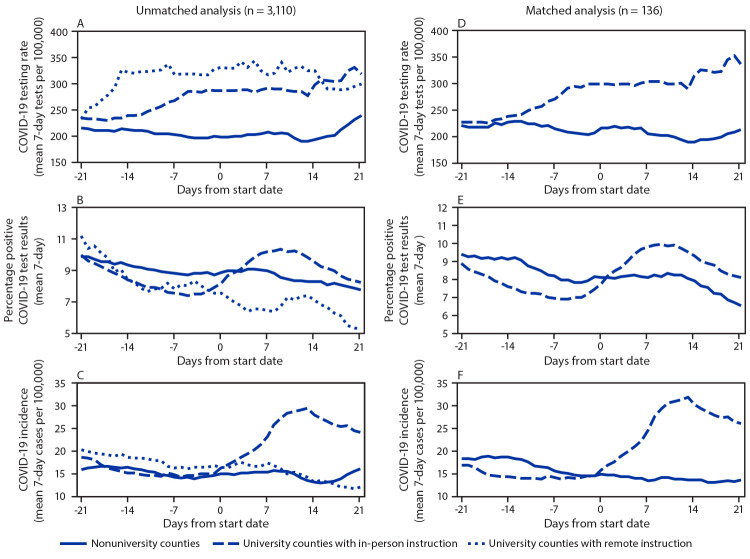
Trends* in COVID-19 testing rates (A, D), percentage test positivity (B, E), and incidence (C, F) for unmatched U.S. counties^†^ and counties matched^§^ based on population size and geographic proximity, 7-day moving average — United States, 2020 **Abbreviation:** COVID-19 = coronavirus disease 2019. * Trends are presented relative to the start date for fall 2020 classes for counties with large colleges and universities (university counties) and the assigned start date for nonuniversity counties. ^†^ University counties with remote (n = 22) and in-person (n = 79) instruction versus nonuniversity (n = 3,009) counties. ^§^ University counties with in-person instruction versus nonuniversity counties (68 matched pairs). Matches for each in-person university county were identified by listing all candidate (county) matches without large colleges or universities that had a similar population size (± 30%) and that were located within 500 miles (805 km) of each university county. From these candidate matches, the final match was selected based on closest proximity such that no nonuniversity county was matched more than once. After matching, the average distance between counties in matched in-person university county and nonuniversity county pairs was 114 miles (183 km) with a maximum distance of 471 miles (758 km). Eleven in-person university counties were excluded from the matched analysis because there were no candidate matches meeting population size and proximity specifications. All remote university counties were excluded from the matched analysis because there was an insufficient number of nonuniversity county matches.

COVID-19 outcomes were similar in the matched analysis. Compared with nonuniversity counties, in-person instruction university counties experienced a higher relative change in testing rates (18.8% versus –5.6%), a higher absolute change in test positivity (1.6% versus –0.8%), a higher relative change in incidence (78.3% versus –19.5%) (Table) (Figure), and a higher absolute change in the percentage identified as hotspots (33.8% versus 1.5%). Based on the difference-in-difference analysis, university counties with in-person instruction were associated with an increase of 14.4 cases per 100,000 (p<0.05) and an increase of 2.4 percent test positivity (p<0.05) relative to nonuniversity counties with in-person instruction. When adjusting incidence for the influx of full-time students, in-person instruction university counties were associated with an increase of 10.6 cases per 100,000 (p<0.05) (Supplementary Table, https://stacks.cdc.gov/view/cdc/99533). These results did not change meaningfully in the sensitivity analysis.

## Discussion

County-level COVID-19 incidence decreased in much of the United States in late summer 2020. Comparing the 21 days before and after instruction start dates, university counties with in-person instruction experienced a 56% increase in incidence and 30% increase in hotspot occurrence as well as increases in COVID-19-related testing and test percentage positivity. Results from the unmatched analysis were consistent with those from the matched analysis. If percentage positivity had been stable or declining across the observation period, then efforts on the part of many colleges and universities to conduct or require testing before students’ return to campus and their ongoing surveillance efforts might explain an increase in case counts, as a result of increased case discovery. However, the concurrent increases in percentage positivity and in incidence in these counties suggest that higher levels of transmission, in addition to increased case discovery, occurred in these communities (*2*).

The findings in this report are subject to at least six limitations. First, data abstraction for schools’ instructional formats was conducted in early September and focused on identifying the format used on the first day of classes; some misclassification of instructional format might have occurred because of changes during the first few weeks of instruction. Second, this study did not adjust for mitigation strategies (e.g., mask and social distancing requirements and limits on large crowds and athletic events) implemented at local or state levels or at colleges and universities, which could have affected the association between the institution’s opening and county-level incidence. Similarly, whether cases in university counties were college- or university-related (i.e., through contact in classrooms, dormitories, cafeterias, or off-campus activities) or related to community transmission could not be discerned. Third, these results might not be generalizable to counties with smaller colleges and universities. Fourth, U.S. Census 2019 population estimates were used to calculate rates, which do not include all college and university enrollments. County-level rate calculations could be inflated for university counties, especially those for which the enrollment numbers are relatively large compared with the county’s population size. Fifth, the longer-term implications for county incidence (i.e., beyond 21 days) were not assessed. Finally, the university counties in the unmatched analysis have larger populations and likely additional characteristics that are different from those of nonuniversity counties. This limitation prompted the decision to conduct the matched analysis, which focused on counties with more similar population levels and geographic proximity. However, broader generalizations based on the matched analysis might not be warranted because 11 university counties with in-person instruction were excluded from the matched analysis because no appropriate matches were available.

COVID-19 incidence, hotspot occurrence, COVID-19-related testing, and test positivity increased in university counties with in-person instruction. Efforts to prevent and mitigate COVID-19 transmission are critical for U.S. colleges and universities. Congregate living settings at colleges and universities were linked to transmissions (*7*). Testing students for COVID-19 when they return to campus and throughout the semester might be an effective strategy to rapidly identify and isolate new cases to interrupt and reduce further transmissions (*8*). Colleges and universities should work to achieve greater adherence to the recommended use of masks, hand hygiene, social distancing, and COVID-19 surveillance among students (*9*), including those who are exposed, symptomatic, and asymptomatic. The increase in testing rates likely reflects local efforts already underway to improve COVID-19 surveillance and response. Increasing testing capacity and engaging in other COVID-19 mitigation strategies might be especially important for colleges and universities in areas where transmission from students into the broader community could exacerbate existing disparities, including access to and utilization of health care, as well as the disproportionate morbidity and mortality of COVID-19 among populations with prevalent underlying conditions associated with more severe outcomes following infection. Some university counties might have one or more concerning factors, such as higher levels of older adult populations, high rates of obesity and cardiovascular disease, or strained health care resources. These counties might need to consider the implications of in-person instruction on spread of COVID-19 among a student population that might have interactions with persons at higher risk in the community. College and university administrators should work with local decision-makers and public health officials to strengthen community mitigation, in addition to continuing efforts to slow the spread of COVID-19 on college and university campuses.

**Summary**

**What is already known about this topic?**

Increasing COVID-19 incidence was observed among young adults in August 2020, and outbreaks have been reported at institutions of higher education (colleges and universities).

**What is added by this report?**

U.S. counties with large colleges or universities with remote instruction (n = 22) experienced a 17.9% decrease in incidence and university counties with in-person instruction (n = 79) experienced a 56% increase in incidence, comparing the 21-day periods before and after classes started. Counties without large colleges or universities (n = 3,009) experienced a 6% decrease in incidence during similar time frames.

**What are the implications for public health practice?**

Additional implementation of effective mitigation activities at colleges and universities with in-person instruction could minimize on-campus COVID-19 transmission and reduce county-level incidence.

## Footnotes

* Instructional format was assigned based on the advertised method of instruction for the first day of fall 2020 classes. “Remote” format was defined as an instructional format that appeared to minimize in-person classwork on campus. This definition did allow in-person instruction for a very select number of students, including those in laboratory courses, studio courses, or courses for small groups of students with specific instructional needs. In contrast, the “in-person” format was defined for all other colleges and universities that were not considered remote, which included any instructional format that did not appear to minimize in-person classwork on campus. “Hybrid” instructional formats that had reduced, but reoccurring, in-class experiences for many college and university courses (i.e., beyond laboratory and studio courses) were considered “in-person” for this study. The assignment of instructional format was based on the advertised method of instruction and was not based on the college or university policy toward on-campus housing; therefore colleges and universities with remote instruction could have allowed students to stay in on-campus housing.

† A total of 149 large colleges and universities were identified across 133 counties.

§ Incidence was calculated using COVID-19 case counts from state and county health department websites compiled by USAFacts (https://usafacts.org/).

¶ County-level testing rates and rates of percentage positivity represent viral COVID-19 laboratory diagnostic and screening test (RT-PCR) results and exclude antibody and antigen tests. COVID-19 Electronic Laboratory Reporting state health department-reported data are used to describe county-level RT-PCR result totals when information is available on patients’ county of residence or health care providers’ practice location. HHS Protect laboratory data (provided directly to the federal government from public health laboratories, hospital laboratories, and commercial laboratories) are used otherwise. Total RT-PCR tests reflect the number of tests performed, not the number of persons receiving testing. RT-PCR test positivity rate is the number of positive tests divided by the total number of tests performed and for which results were available.

** Hotspot , or rapid riser, counties met all four of the following criteria, relative to the date assessed: 1) >100 new COVID-19 cases in the most recent 7 days; 2) an increase in the most recent 7-day COVID-19 incidence over the preceding 7-day incidence; 3) a decrease of no more than 60% or an increase in the most recent 3-day COVID-19 incidence over the preceding 3-day incidence; and 4) a ratio of 7-day incidence to 30-day incidence exceeding 0.31. In addition, hotspots must have met at least one of the following criteria: 1) >60% change in the most recent 3-day COVID-19 incidence or 2) >60% change in the most recent 7-day incidence. CDC and other federal agencies that are monitoring trends in COVID-19 are collaborating to refine approaches to define and monitor hotspots. As a result, terminology or definitions used in future reports might differ from those used in this report.

†† Matches for each in-person university county were identified by listing all candidate (county) matches without large colleges or universities that had a similar population size (± 30%) and that were located within 500 miles (805 km) of each university county. From these candidate matches, the final match was selected based on closest proximity such that no nonuniversity county was matched more than once. After matching, the average distance between counties in matched in-person university county and nonuniversity county pairs was 114 miles (183 km) with a maximum distance of 471 miles (758 km). Eleven in-person university counties were excluded from the matched analysis because there were no candidate matches meeting population size and proximity specifications. All remote university counties were excluded from the matched analysis because there were an insufficient number of nonuniversity county matches.

§§ Difference-in-difference is a statistical technique that compares the changes in outcomes over time between two groups: those who are part of a control group and those who are part of a treatment or an intervention group. In this analysis, the intervention group was considered to be the counties with colleges and universities that had in-person instruction and the control group was considered to be nonuniversity counties. Difference-in-difference estimates used a regression model with the following specification: Y_ct_ = α + β_1_∙In Person_ct_ + β_2_∙After_ct_ + δ_IP_∙After_ct_∙In person_ct_ + θ_c_ + θ_s_ + θ_week_ + θ_weekday_ + ε_ct_, where Y_ct_ is the outcome of interest (i.e., either COVID-19 incidence or percentage test positivity) for each county *c* and each unit of time *t* (days); In Person_ct_ is an indicator equal to 1 if the county has a college or university that started classes in an in-person format; After_ct_ is an indicator equal to 1 for all the days after the county's assigned start date (i.e., an indicator equal to 1 for days 0 to 21, where day 0 is the start date); θ_c_ and θ_s_ are county- and state-level fixed effects; θ_week_ and θ_weekday_ are fixed effects for each calendar week and each weekday; and ε_ct_ is the unobserved error term. The coefficient of interest is δ_IP_, which captures the difference in outcome before and after the start date among in-person university counties, minus the difference in outcome before and after the assigned start date in nonuniversity counties. Standard errors were clustered at the county level. A placebo test was conducted where the college or university start date used day –21 as the demarcation of before and after periods, and no violation of the parallel trends assumption was found.

¶¶ Because transient student populations might not be included in the population denominator for county incidence estimates, incidence is assessed two ways in the difference-in-difference models: first using county population reported by the U.S. census, then adjusting for student influx by adding full-time student enrollment to each college or university’s county population for the period after classes start. The full-time student population was used for this adjustment instead of the total student population, which includes full-time and part-time students.
